# Complexity and involvement as implementation challenges: results from a process analysis

**DOI:** 10.1186/s12913-021-07090-z

**Published:** 2021-10-23

**Authors:** Yvette Emond, André Wolff, Gerrit Bloo, Johan Damen, Gert Westert, Hub Wollersheim, Hiske Calsbeek

**Affiliations:** 1 Scientific Center for Quality of Healthcare (IQ healthcare), Radboud Institute for Health Sciences (RIHS), Radboud university medical center, PO Box 9101, 114 IQ healthcare, 6500 HB Nijmegen, The Netherlands; 2 Department of Anesthesiology, Pain and Palliative Care, Radboud university medical center, Nijmegen, The Netherlands; 3 Department of Anesthesiology, Pain Center, University of Groningen, University Medical Center Groningen, Groningen, The Netherlands

**Keywords:** Guideline adherence, Implementation, Multifaceted approach, Patient safety, Perioperative care, Stepped-wedge design

## Abstract

**Background:**

The study objective was to analyse the implementation challenges experienced in carrying out the IMPROVE programme. This programme was designed to implement checklist-related improvement initiatives based on the national perioperative guidelines using a stepped-wedge trial design. A process analysis was carried out to investigate the involvement in the implementation activities.

**Methods:**

An involvement rating measure was developed to express the extent to which the implementation programme was carried out in the hospitals. This measure reflects the number of IMPROVE-implementation activities executed and the estimated participation in these activities in all nine participating hospitals. These data were compared with prospectively collected field notes.

**Results:**

Considerable variation between the hospitals was found with involvement ratings ranging from 0 to 6 (mean per measurement = 1.83 on a scale of 0–11). Major implementation challenges were respectively the study design (fixed design, time planning, long duration, repeated measurements, and data availability); the selection process of hospitals, departments and key contact person(s) (inadequately covering the entire perioperative team and stand-alone surgeons); the implementation programme (programme size and scope, tailoring, multicentre, lack of mandate, co-interventions by the Inspectorate, local intervention initiatives, intervention fatigue); and competitive events such as hospital mergers or the introduction of new IT systems, all reducing involvement.

**Conclusions:**

The process analysis approach helped to explain the limited and delayed execution of the IMPROVE-implementation programme. This turned out to be very heterogeneous between hospitals, with variation in the number and content of implementation activities carried out. The identified implementation challenges reflect a high complexity with regard to the implementation programme, study design and setting. The involvement of the target professionals was put under pressure by many factors. We mostly encountered challenges, but at the same time we provide solutions for addressing them. A less complex implementation programme, a less fixed study design, a better thought-out selection of contact persons, as well as more commitment of the hospital management and surgeons would likely have contributed to better implementation results.

**Trial registration:**

Dutch Trial Registry: NTR3568, retrospectively registered on 2 August 2012.

**Supplementary Information:**

The online version contains supplementary material available at 10.1186/s12913-021-07090-z.

## Background

From 2010 to 2013, national perioperative guidelines (including Patient Safety Indicators [PSIs]) were introduced into all hospitals in the Netherlands to improve perioperative patient safety [1–3]. The perioperative safety guidelines cover the full perioperative trajectory; from preoperative surgical and anaesthesia risk-assessment, patient admission, and surgical procedure to patient discharge from the hospital. Properly used, the guidelines should ensure that critical tasks are carried out and that the team and patient are adequately prepared for the next step in the trajectory. The process implies that everyone in the team has a responsibility to communicate and to speak up if they foresee or notice any errors or problems, in other words they should have an equal position, avoiding hierarchy between team members.

Implementation of a guideline can be defined as the planned and systematic introduction, aiming to integrate its recommendations into professional actions [4]. Effective implementation of perioperative patient safety guidelines should ensure guideline adherence in practice and subsequently lead to improved patient outcomes and safety [5]. Studies revealed that better guideline compliance is associated with better perioperative outcomes [6, 7]. Therefore, adherence to the perioperative safety guidelines is an important target for perioperative safety improvement. Consequently, implementation interventions need to be identified that are appealing to the target group. Research studies on the effectiveness of different interventions have, however, shown that no strategy is superior in all situations, most are useful in some settings [8, 9] and multifaceted interventions do not naturally yield more effect than single ones [10, 11]. However, interventions tailored to prospectively identified barriers and facilitators are more likely to improve professional practice as compared with non-tailored interventions [9, 12–14].

The IMPROVE-implementation programme was developed based on an extensive analysis of the barriers and facilitators for the implementation of the national perioperative safety guidelines [15]. To evaluate the impact of the IMPROVE-implementation programme on the safety of perioperative care (see Additional file 1), we conducted a stepped-wedge cluster-randomized trial in nine hospitals (three groups of three hospitals each) in the Netherlands (Emond et al.: Increased adherence to the national perioperative safety guidelines associated with improved patient safety outcomes. Results of the IMPROVE implementation study, a stepped-wedge, cluster-randomized multicentre trial, submitted). The evaluation included 1934 high-risk surgical patients undergoing elective abdominal or vascular surgery with a mortality risk ≥1% (exclusion criteria were: < 18 years; day-care (hospital admissions of ≤24 h); cardiac surgery; organ transplantations (except kidney transplants); and emergency surgery) and showed some improvements over time, such as increased guideline adherence (between 7 and 30 percentage points), decreased postoperative wound infections (from 13.6 to 2.6%) and decreased length of hospital stay (from 8 to 6 days). However, most effects were not significant or related to the implementation programme, probably due to heterogeneous implementation success (Emond et al.: Increased adherence to the national perioperative safety guidelines associated with improved patient safety outcomes. Results of the IMPROVE implementation study, a stepped-wedge, cluster-randomized multicentre trial, submitted). It is important to better understand this implementation process in the different perioperative contexts of the hospitals included, as guidelines will continue to be (further) developed and need to put into practice to optimise the quality and safety of care [16]. For this reason, we aimed to explore the involvement in the implementation activities.

Even though much emphasis is always placed on the effect evaluation to determine whether a programme is successful, a process evaluation helps to understand why the programme was or was not successful, which is equally important [17, 18]. A process evaluation can illustrate the mechanisms and processes responsible for the results and their variation within target groups. It provides information about the strategy as planned and as delivered and about exposure of participants to the implementation activities and experience of those exposed. Many interventions aimed at improving healthcare and patient outcomes are complex in the sense that they are composed of several interacting components [19]. Randomized controlled trials of such interventions are often criticized as being ‘black boxes’, since it can be difficult to know why the intervention worked (or not) without examining underlying processes. Process evaluations are recommended to open the ‘black boxes’ of complex interventions evaluated in trials and are considered essential in complex implementation programmes. In multisite trials, the ‘same’ programme may be implemented in different ways. Process evaluations can be used to provide insights into what extent the implementation programme was actually implemented and how it was experienced. A strategy for change can only have its theoretical impact if it is implemented as intended by its developers [20]. Including a process evaluation in research is especially necessary in multisite studies, where the same strategy may be implemented to different degrees and in different ways.

This article describes the results of the process analysis approach (PAA) based on the following research questions:

1. What was the degree of involvement of the target group in the IMPROVE-implementation programme, and did the hospitals involved differ in this regard?
2. What challenges arose while introducing the IMPROVE programme?

## Methods

### The IMPROVE-implementation programme

The IMPROVE-implementation programme involves a multifaceted intervention within the perioperative setting, tailored to: local barriers identified prospectively in the participating hospitals [15]; current performance and guideline adherence in the hospitals; and local needs and initiatives already realized or planned in the hospital. It uses evidence from scientific literature (systematic review of interventions by Grimshaw et al. [8]); expert opinion (perioperative healthcare professionals as well as implementation experts [*N* = 13 and 11 experts respectively, there was no overlap between groups] were asked to rate potential interventions in terms of their usefulness in improving perioperative guideline adherence) and knowledge and experience of the research team regarding the feasibility of the interventions (estimated costs, effort, and time for the hospitals as well as the research team) [21]. Perioperative healthcare professionals covered the full range of perioperative disciplines and had ample experience in the perioperative field and an interest in the implementation of the perioperative safety guidelines. Implementation experts were persons with different work settings and specialties, all with ample experience and working in the field of implementation science in healthcare. Overall, the experts had 20.9 ± 9.5 years work experience in their field of perioperative care or implementation science. The standard components of the implementation programme included: small educational meetings; audit & feedback (based on local indicator scores, benchmarks and barriers); structured observation rounds with feedback; integration of the guideline recommendations in (existing) local activities and processes; and the use of patient safety cards. A set of six additional activities were offered as being optional [21]. See also Additional file 2.

A structured programme-implementation approach was used (see Table 1). At the start of a new intervention phase, we organized a kick-off masterclass for the target group of three hospitals in the stepped-wedge trial. Per hospital, we invited a manager (someone from the hospital at the highest level), a local expert with knowledge of improvement and change management (e.g. research manager, quality & safety manager), and a clinical champion, i.e. a medical specialist as a role model within the perioperative care trajectory. These people were intended to play a key role in the implementation activities in their hospital, next to the contact person(s) who were selected by the hospitals themselves during the selection process of the participating hospitals. After the kick-off meeting, we visited the hospitals to concretize the execution of the implementation programme. During the study, we sent four newsletters to the contact person(s) for further internal distribution. To check whether the extracted data concurred with the original patient administrative data, a quality check of the data was performed by data managers using a random selection of five cases per hospital per measurement. The data managers could be any person with knowledge about the data storage within the hospital. They helped us with the retrieval of the data. These persons were designated by the contact persons. No selection criteria were used.

**Table 1:** The structured programme implementation approach. All hospitals received structured support to start and sustain the intervention phase

Masterclass^a^	Planning interview^b^	Extra visits	Newsletters	Data control
• Introduction to the IMPROVE study • Interactive presentations about perioperative safety awareness, the content of the perioperative safety guidelines and implementation strategies • Exchange of local intervention activities • Brainstorming, input of own ideas (and wishes) • Presenting and explaining the implementation activities manual • Room for Q&A	• Local visits to discuss chosen implementation activities and work out the planning • Report with agreements on intended activities	• If necessary to discuss progress and adjustments	• Inform hospitals about the study (progress and interim results)	• Hospitals collect the data of 5 cases per measurement • These data were compared with the data we collected • Results were fed back to the hospital

^a^ Prepared and conducted by the research team

^b^ Planned and conducted by the researcher together with one or more members of the research team

### Process analysis approach

In our implementation programme we developed a process evaluation questionnaire to assess the participants experiences with the individual implementation activities and the overall IMPROVE programme. However, the low involvement in the programme was also reflected in the response on the questionnaire. In line with the low involvement in the IMPROVE programme, participants insufficiently (2 contact persons out of 11) filled in the surveys. For this reason, the questionnaire results could not be used for a standard PAA. In turn, we developed an alternative PAA that measures implementation involvement by an involvement rating measure and field notes.

#### Degree of involvement – involvement rating measure

The new measure reflects the number of activities in combination with the degree of involvement per activity (i.e. the extent to which hospitals executed the IMPROVE manual for implementation activities), based on the researchers’ observations and reports from the contact persons in the hospitals as monitored in the field notes (see below). For the five standard activities, hospitals could receive 0 to 2 points per activity or 3 points in case of the audit & feedback activity based on their involvement per activity during the intervention period (Table 2). The overall involvement rating score (total accumulated score) per hospital thus ranged from 0 to 11 (four activities with a maximum of 2 points and one activity with maximum 3 points). We considered 0–3 points as unsatisfactory involvement, 4–7 points moderate and 8–11 points as satisfactory involvement.

**Table 2:** Description of the involvement rating scoring

IMPROVE-implementation activity	Involvement score	Explanation
Small educational meetings		
	0	No educational meetings
	1	The educational meeting partly took place according to the manual
	2	The educational meeting fully took place according to the manual
Audit & feedback		
	0	No audit and feedback
	1	The hospital received the feedback report before the next intervention phase
	2	The feedback meeting partly took place according to the manual
	3	The feedback meeting fully took place according to the manual
Structured observation rounds		
	0	No structured observation
	1	An observation round by a trained (external) expert with feedback based on a structured observation list. Hospitals received the observation tool for own use.
	2	Observation rounds by own hospital personnel based on the structured observation list
Integration of the guideline recommendations		
	0	No integration of guideline recommendations in local activities and processes
	1	Integration of 1 recommendation in local activities and processes
	2	Integration of 2 or more recommendations in local activities and processes
Use of patient safety cards		
	0	No use of patient safety cards
	1	Patient safety cards were offered
	2	Patient safety cards were used (with the explicit invitation by caregivers to patients to ask questions)

It was shown that the involvement rating was positively related to implementation of the STOP bundle (composite outcome defined as the percentage of patients in which all the stop moments have been performed) as well as three (out of six) separate stop moments of the STOP bundle: the time-out, discharge from the recovery ward, and discharge from the hospital (all *P* < .001) (Emond et al.: Increased adherence to the national perioperative safety guidelines associated with improved patient safety outcomes. Results of the IMPROVE implementation study, a stepped-wedge, cluster-randomized multicentre trial, submitted), meaning that these outcomes (i.e. adherence to these PSIs) improved as the degree of involvement in the IMPROVE programme increased.

#### Implementation challenges – field notes

During the study period, we prospectively kept a logbook to keep track of our “implementation” experiences in the hospitals in order to explain the involvement rating and to identify challenges for carrying out the IMPROVE study. The logbook contained the notes of all meetings and contacts with the hospitals (including all mail exchange), descriptions of the key features of performed implementation activities (e.g. target group, implementer, intensity) based on the framework of Hulscher et al. [22] as well as attendance logs and, per hospital, a schedule with the planning and distribution of tasks and responsibilities (including to do’s, deadlines and the current state of affairs) based on the planning interview. Chiseri-Strater and Sunstein [23] have developed a list of what should be included in field notes for anthropology. For example; date and place of observation; specific facts, numbers, details of what happens at the site and who is involved; specific words, phrases, summaries of conversations, and insider language. We documented and structured our notes and current state of affairs of the IMPROVE activities in the planning schedule according to this list.

**Fig. 1:**
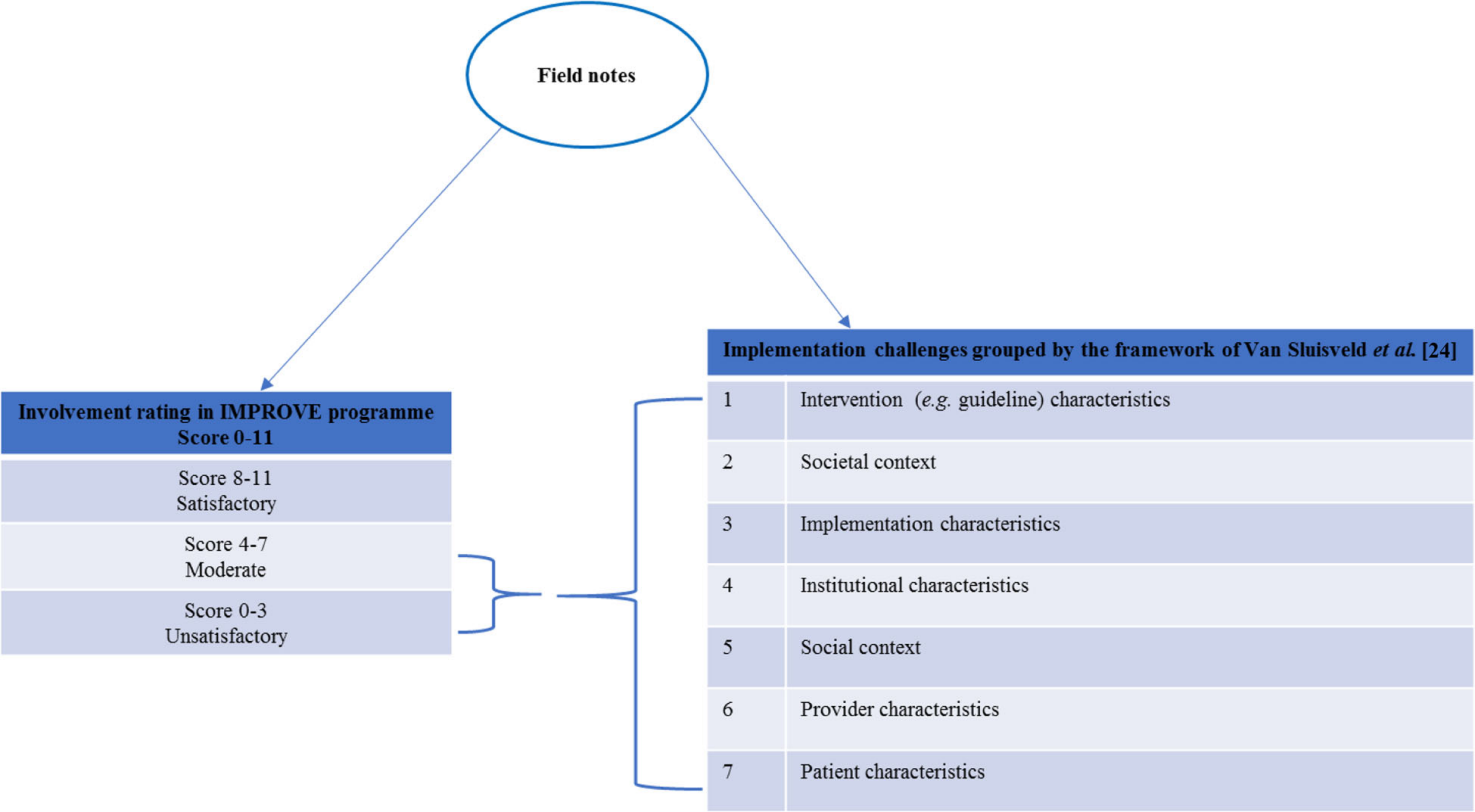
The process analysis method used in this study. In Fig. 1, the process analysis method is summarized: during the study period, we prospectively kept all field notes in order to explain the involvement rating and to identify challenges in carrying out the IMPROVE study. In case of a moderate or unsatisfactory involvement rating score, we looked at the implementation challenges as reported in the field notes in order to explain these low ratings

The research team discussed the identified challenges and classified them into the barrier categories of the framework of Van Sluisveld et al. [24]. This framework helps to provide insight into the implementation process, as well as into factors influencing this process. This framework is based on three models related to implementing change: the implementation of change-model of Grol and Wensing [25, 26]; the framework of knowledge–attitude–behaviour-related barriers for guideline adherence of Cabana et al. [27]; and the framework for adherence to clinical practice guidelines in the intensive care unit of Cahill et al. [28]. We used the framework of Van Sluisveld et al. also for our barrier analysis, prior to the actual implementation of the perioperative guidelines [15]. The categories of the framework van Van Sluisveld et al. [24] relate to: 1. intervention characteristics (e.g. complexity and feasibility of the guidelines); 2. the societal context (e.g. legal obligations and regulations); 3. implementation characteristics (e.g. exposure to implementation efforts); 4. institutional characteristics (e.g. organizational structure, time, [financial] resources, equipment, IT structure); 5. the social context (e.g. behaviour of colleagues, collaboration, culture in the team); 6. provider characteristics (e.g. their motivation, opinions, attitudes, behavioural routines, habits, expectations); or 7. patient characteristics (e.g. their preferences) (Fig. 1).

## Results

### Degree of involvement

The planned IMPROVE interventions were performed with varying degrees of involvement in the hospitals. Involvement in the implementation programme was low to moderate, with involvement rating scores ranging from 0 to 6 (on a scale of 0–11) and a mean score of 1.83 per measurement period. The programme worked in a somewhat greater degree in two hospitals (hospital A and C) in the first group of the stepped-wedge trial (Table 3).

**Table 3:**
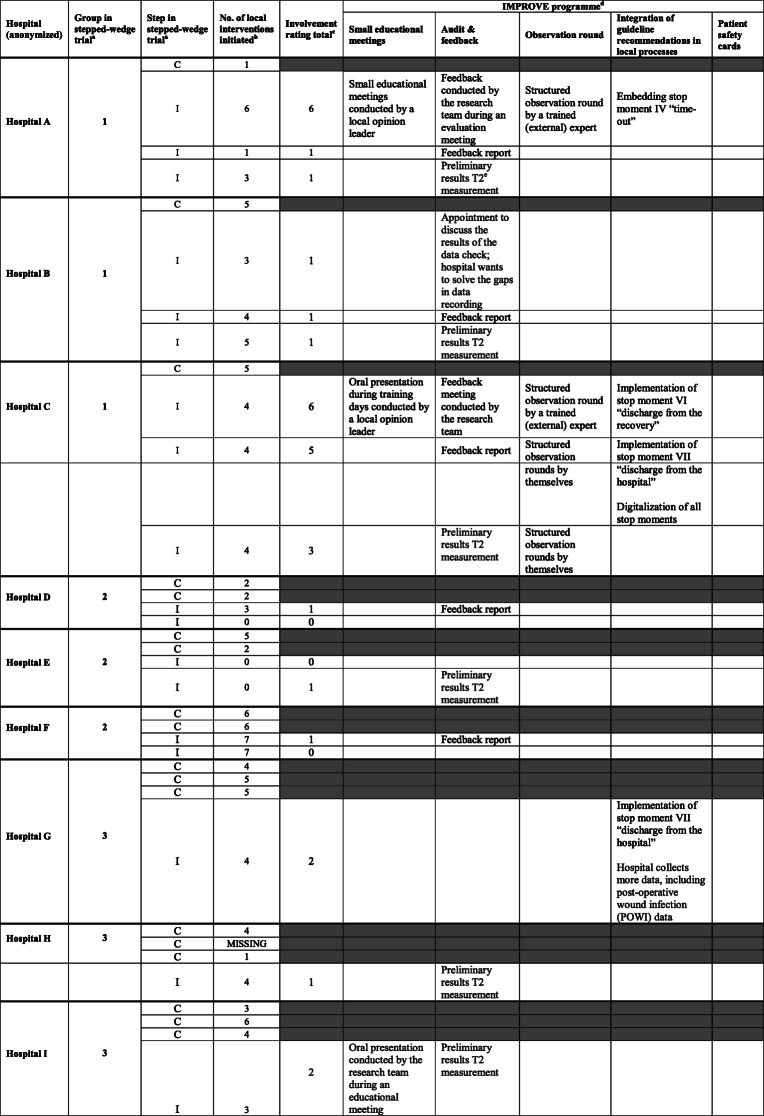
Characteristics of implementation activities

^a^ Stepped-wedge design with three groups of three hospitals each, and four steps in which “C” represents the Control situation and “I” the Intervention phase

^b^ Co-interventions additional to IMPROVE

^c^ Hospitals’ involvement in the IMPROVE-implementation programme. For the five standard activities, hospitals could receive 0 to 2 points per activity or 3 points in case of the audit & feedback activity based on their involvement per activity. The overall involvement rating score (total accumulated score) per hospital thus ranged from 0 to 11. We considered 0–3 points as unsatisfactory involvement, 4–7 points moderate and 8–11 points as satisfactory involvement

^d^ The five standard activities of the implementation programme

^e^ T2 = measurement at T2. In total, there were four measurements (T0-T3). T0 is the baseline measurement (no hospital received the intervention), T1 is the second measurement (when three hospitals received interventions), T2 is the third measurement (when six hospitals received interventions), and T3 is the final measurement (when all nine hospitals received interventions)

Small educational meetings (with different designs and a varied composition of target professionals) and the integration of guideline recommendations in local activities and processes were the most-used implementation activities, followed by feedback meetings as well as structured observation rounds with feedback. No hospital used the patient safety cards (Table 3).

### Implementation challenges

**Fig. 2:**
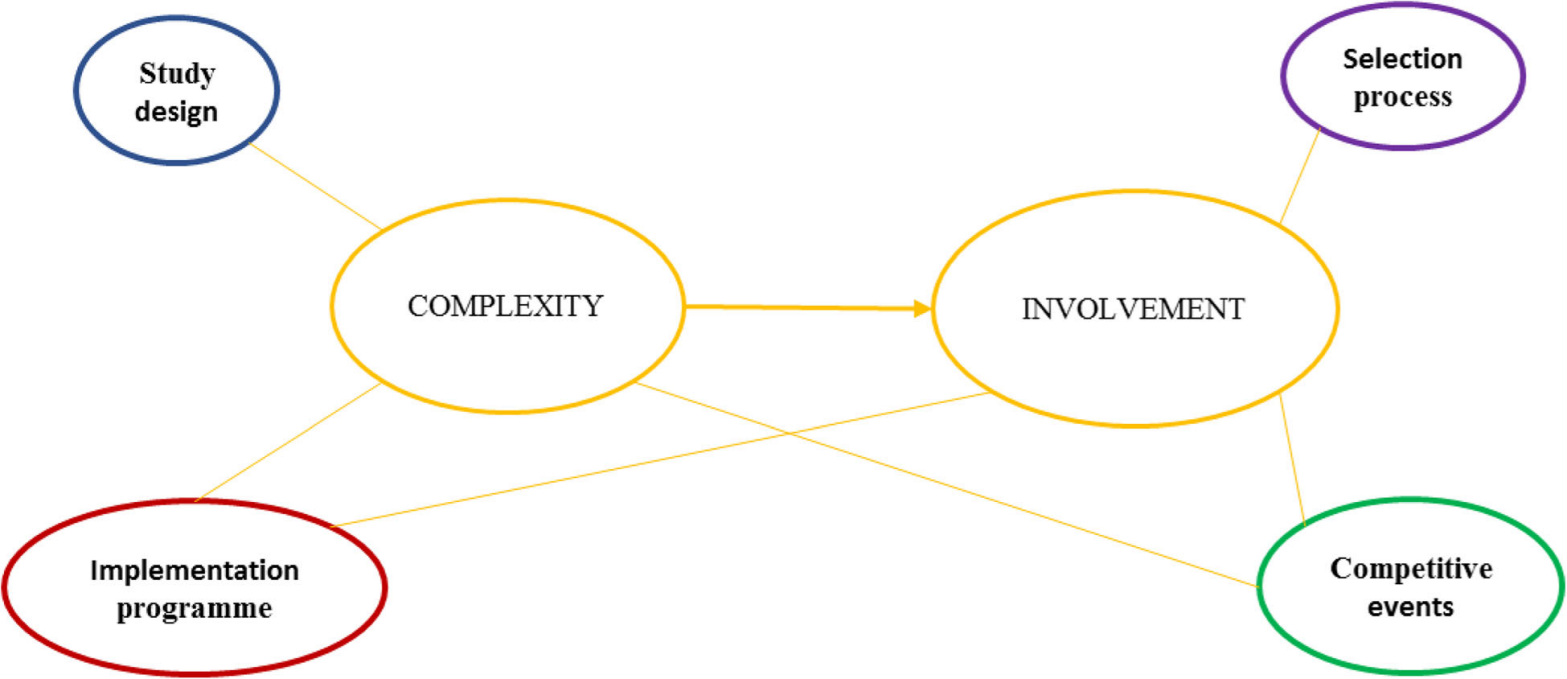
Overview of the implementation challenges reflecting a high complex intervention and factors that seriously affected the involvement of professionals. The field notes yielded four major implementation challenges: study-design complexity; intervention and implementation complexity, i.e. the implementation programme; the selection process of hospitals, departments and key contact person(s) (inadequately covering the entire perioperative team and stand-alone surgeons); and competitive events. These implementation challenges induced and reinforced each other

The field notes yielded four major implementation challenges, reflecting a high complex intervention and factors that seriously affected the involvement of professionals (see Fig. 2 and Table 4). These challenges related to the study design, the selection process, the implementation programme, and competitive events. Using the framework of Van Sluisveld et al. for the implementation of guidelines and interventions (see Fig. 1), most challenges related to implementation characteristics, such as: the study design (stepped-wedge); the selection process of the hospitals, departments and contact person(s); and the implementation programme (e.g. tailoring, multicentre). Competitive events and local intervention activities appeared to be important challenges related to institutional characteristics. In addition, there were three challenges related to the societal context (i.e. co-interventions by the Inspectorate), the social context (i.e. stand-alone surgeons) and provider characteristics (i.e. complexity of the perioperative team). The Dutch Health Care Inspectorate (IGJ) has been monitoring the safety of the surgical process since 2006 in their programme “Supervision of the surgical process” (in Dutch: Toezicht Operatief Proces [TOP] 1, 2 and 3) [29–31]. See also Additional file 3.

**Table 4:** Overview of the challenges while introducing the IMPROVE programme with a direction for solutions

Implementation challenge	Content of the challenge	Suggested solutions to the challenge
*Study design*	• Fixed periods • Planning – step length; intervention and measurement period too short - Delayed start of intervention - Feedback data not analysed on time - Patient inclusion too slow • Long trial duration • Repeated measures – data amount needed & collection puts heavy burden on researchers	• Consider a less complex, more flexible study design such as the time series design • Increased step length • Investigating needed implementation support and offering additional support
	• Data availability - No standardization in registration systems - Data not readily available or not routinely collected	• Pilot measurement • Documentation of data sources • Verification of data sources • Data available, reliable, valid and seen in relation to the burden of collecting • Request support when using data management systems, involve for example someone from IT in collecting the data
*Selection process*	Participating hospitals and departments	
	• Recruitment based on personal contacts, often in higher hierarchical levels • Top-down decision to participate • Low implementation involvement • Insufficient sense of urgency • No strong leadership • Insufficient management support - Focused on perioperative setting and insufficiently involved higher management	• Improvement culture • Recruitment on volunteer base • Bottom-up project approach • Joint decision to participate • Sense of urgency • Effort to create local commitment and internal instead of external motivation • Involvement of the higher management before the start of the study • Project fits the mission and objectives of the hospital • The Executive Board and department head should be sponsors of the programme • Ask the department head and a member of the Executive Board to sign the report with agreements on intended activities
	• Lack of financial resources hospital/departments	• Financial resources
	The contact persons	
	• Lack of influence of contact persons • Wrong contact persons with too limited mandate • In five hospitals, one contact person, and in one hospital no contact person during last intervention period • Dependent on agenda and prioritization by contact person	• Carefully think about their functions, internal roles and knowledge domains • Multiprofessional stakeholder group with one key person per discipline (including surgeons) who could also function as a sounding board group • More contact persons can share tasks
	Perioperative team	
	• Target group insufficiently informed and involved in participation decision	• Information meetings to inform the target group periodically about the progress, barriers and results of the programme and let them contribute ideas • Information transfer via colleagues, not only research team • Willingness to change • Study should be accepted by target group • Ownership of the implementation programme • The programme is visible in the agenda of meetings
	• Multiprofessional • Professional cultures • Hierarchical context • Cover parts of the process chain	• Acknowledgement of the cultures, hierarchies and responsibilities within the involved perioperative disciplines
	• Lack of time	• Efficient alterations in time allocation
	Stand-alone surgeons	
	• Social culture: surgeons did not consider themselves as part of the target group	• Surgeon cooperation • Representation of surgeons in the research team or as contact person
*Implementation programme*	• Three guidelines	• A less ambitious implementation programme
	• Extensive implementation programme • Multicentre • Local tailoring • Insufficient mandate of the research team - Hospitals made own intervention choices - Hospitals did not want to execute certain activities or no realization through financial and practical barriers - Some IMPROVE activities were already realized, such as observation rounds and patient safety cards	• Good information in advance • Members of the research team are representative of the target group and experts in the concerning field • Adequate programme budget
	• Local interventions competed for time and money • Time schedule stepped-wedge design did not always correspond with local planning of own interventions • Intervention fatigue and pressure due to other obligations	• Coordination of own activities and those in study context; steering and prioritizing them
	• Co-intervention by Inspectorate - Unexpected time/location - No deliberation/co-planning or coordination	• Good and structured documentation of the co-interventions
*Competitive events*	• Hospital mergers • Digital innovation • Formal/top down pause of activities • Accreditation process	• Beyond our influence; as a research team you have no influence on whether or not competitive events are present

In summarizing these implementation challenges in Table 4 we also suggest concrete solutions to address these challenges based on our experiences in this study and what actually worked in two hospitals.

## Discussion

### Main findings from this study

Due to the complexity of the programme, the context in which it was implemented and the many factors that affected the (internal) motivation of the target professionals, their involvement was suboptimal. This varied from poor to moderate: two out of nine hospitals were moderately active in executing the IMPROVE-implementation activities during one or two steps in the stepped-wedge trial. We had to deal with resistance or – on the contrary – with a very promising start in some hospitals, who had huge plans that, however, mostly stranded in practice. Four major implementation challenges were derived from our field notes: the study design; the selection process of hospitals, departments and key contact person(s) who inadequately represented the entire perioperative team and stand-alone surgeons; the implementation programme; and competitive events. We will now subsequently discuss each of these four challenges.

### Interpretations and comparison with literature

#### The study design

A huge challenge was our choice for the stepped-wedge design. This design turned out to be less suitable for this type of research in practice. Different aspects hampered the study, such as the fixed timing of the intervention that could not be adjusted and the lack of influence on co-interventions and competitive events with a large impact (audits by the Inspectorate, local interventions, hospital mergers). The fixed time span between the intervention and the post-intervention measurement did not account for the extra time for the research team to get ready and time for all units in an institution to get on board [32]: full implementation takes more time. In a stepped-wedge design, the time intervals between steps should be long enough for the intervention to be rolled out and become fully effective (and for the outcomes to be measured) [33]. A delay in realizing the (full) effectiveness of the intervention in our study probably resulted from a slower than expected and incomplete rollout of the programme. Further, the stepped-wedge design requires cooperation and commitment from the participating hospitals. Hospitals had to be ready to apply the intervention when the randomization order dictates, but we could not mandate this.

The data-collection problems in the participating hospitals were a prominent challenge. Access to the data needed was difficult and sometimes impossible, which hindered the audit & feedback implementation activities. This resulted in major data collection efforts by the researchers and caused delays. Problems encountered in all participating hospitals were:

- Inability to retrieve some data (e.g. missing data*:* 8% of the administration time point of antibiotic prophylaxis, 11% of the postoperative wound infection rates and 5% of the complication rates).
- Fragmentation of data: data were scattered across various registration systems (one-third of the hospitals used two different registration systems within the perioperative care process).
- Parallel registration: the same clinical information was documented in different registrations (a total of five different indicators was documented in two or more places in four out of nine hospitals). It was not always clear which registration system was the most reliable one. Moreover, data managers sometimes did not know where to find their own data. Hospitals had insufficient insight into their own performance and the quality of their data. Additional file 4 presents an example of parallel registration in which the administration time of antibiotic prophylaxis is documented in three electronic data systems, demonstrating missing and inconsistent data.
- Discrepancies between data within hospitals were due to different registrations in electronic and paper files and different registration in discharge information and registration system (82% of the complications were only listed in the discharge letter to the general practitioner and not in the official complication registration).

These problems caused incomplete and partly invalid data; 1.9% of all the variables we collected over the measurements turned out to remain missing after repeated searches. The data collection also proved to be time consuming (10–30 min per surgical patient on average). As we had been told that all data needed could be retrieved reliably with one push on the button, the extra time investment needed was 76 working days of 8 h (9 hospitals × 50 patients × 20 min = 9000 min = 19 working days × 4 measurements).

#### The selection process of hospitals, departments and key contact person(s)

Involving the target group, especially the medical specialists and the top management of the hospital, is a crucial step. Top managers’ commitment is a key determinant of implementation effectiveness [34–40], stressing the importance of the ‘Board on board’. Hospital leaders are frequently seen as enthusiast at the beginning of a programme, taking the initiative to start the changes, but then delegating the actual implementation. Management is expected to be involved and supportive of frontline staff during improvement initiatives and beyond, such that it is seen as an organizational priority from the outset and all levels of the organization are aligned on a common goal [41]. Failure to maintain enthusiasm can, however, undo the introduced change. In contrast, leadership requires a consistent position and reporting on the intended change. Middle managers’ influence on programme implementation turned out to be limited. We did not succeed to actively involve the persons who were invited to the masterclass during the intervention phase. We have not tackled this problem properly within such a large-scale project. Without clear communication by the hospital management, perspectives drifted apart and resistance towards the programme developed.

We worked mainly with one contact person per hospital (often middle managers), who had to represent all perioperative disciplines. This turned out not to work optimally. An additional problem was that there was not enough knowledge of improvement science at the hospital-management level. We should have involved and informed them better. Our idea was to perform the communication via the contact person. However, our contact persons were not always involved enough in the perioperative teams and were unable to reach everyone. With adequate, enthusiast and motivated contact persons, who are able to bond disengaged disciplines and individuals and preferably also a replacement contact person, the chance to succeed could be much higher.

Interventions that require the active participation of healthcare professionals need a high degree of motivation. It is important to indicate what is expected from stakeholders, i.e. how they can make a positive contribution, to listen carefully to what they say about the improvement initiative, to inform them periodically about implementation progress and to create room for questions [42, 43]. A communication plan can help to structure the communication [42]. When stakeholders have a clear understanding of the need for change and the management expectations they will be more likely to support new improvement initiatives [43]. Otherwise, the programme has no priority for the busy professionals. As opinion leaders, medical specialists can be an important and influential factor in the success of the implementation [44]; when they are involved at a later stage, progress is usually much slower. However, many medical specialists remain unknown and sceptical about improvement initiatives. Medical staff generally had only a vague idea of the IMPROVE programme, and only few had direct experience of some of its components. We had the impression that the implementation of the perioperative safety guidelines sometimes received insufficient priority in some participating hospitals. For example, one hospital had no quality advisor operation room (OR) during the second and third intervention period. That function or field of attention apparently disappeared during the study. The priority of patient safety had to compete with other clinical and organizational priorities. Implementation success also strongly depended on sufficient allocated time to implement the IMPROVE activities in daily care practice. As in other studies [45–47], we identified time constraints and a lack of motivation as factors influencing successful change. The medical staff, often under time pressure themselves, were not easily directed to participate in a time-consuming implementation programme. Busy staff apparently experienced participation in the IMPROVE programme as an additional task.

Making sufficient contact at all layers of the hospital and especially with key figures of the stakeholder analysis is important [42]. Our stakeholder analysis focused on the perioperative setting, insufficiently taking into account the higher level of hospital management and the diversity within the perioperative disciplines involved. Perioperative professionals work together in multi-professional teams. However, the question is whether they function as teams, because they consist of so many different professions (such as nurses, surgeons, anaesthesiologists and ICU employees) and variable persons. The OR is a specific multidisciplinary setting with a heterogeneity of professional cultures and hierarchies [48]. It would not be surprising if this complexity created an environment less conducive to successful implementation. Our programme engaged diverse teams that underwent personnel changes over time, involving surgeons who resisted participating in the implementation programme. This was a major drawback, as surgeons’ commitment is particularly important for successful checklist implementation, as shown by Lingard et al. [49]. Possible explanations for their lack of commitment could be the hierarchical structure, the power dynamics of the hospital environment that privileges surgeons (near-absolute power in the OR; the surgeon orchestrates all activities and no one checks his or her power or reprimands them when they misbehave) and the presence of a traditional surgical culture that tolerates these type of behaviours [50], focus on their preoccupation (“carving”) and a lack of system thinking. Surgeons can be the Achilles heel that causes an OR process or culture to remain stuck in the status quo [51]. Surgeons in our study worked side by side with their perioperative colleagues, but a common goal, cohesiveness and team spirit seemed lacking. According to Cochran et al., [50] surgeons in particular have high rates of disruptive behaviour. Surgery training was thought to attract individuals who aspired high-powered careers and unquestioned authority in a situation that required little empathy or emotional connection with patients [50].

If stakeholders, like surgeons are important for the implementation of innovations, it is sensible to check how they view the project by, for example, inviting them to an information meeting or by talking to them. Thereby carefully listening and taking the resistance seriously and trying to look for a common denominator [42]. Overall, it is important to discuss the personal interests and underlying reasons for professionals’ lack of motivation and commitment by approaching them individually. A facilitating approach is helpful in this, where you ask questions such as ‘what can I do for you in order to convince you to participate in this programme?’. Clearly show that the project is integrated with other things in the department by clarifying how the project fits with other ongoing projects or things that are happening in the department [42]. Change is difficult, even for the best performing healthcare professionals. As processes change, so do shifts, workloads, expectations, and responsibilities. Perceived negative consequences can relate to a fear of change from the status quo. Resistance to change is in many cases not resistance to the proposed change, but a reflection of the psychological process involved in letting go of the past ways of working [52]. It is also important to proactively inform the department management about the programme and ask them to bring the programme to the attention, by the internal distribution of the newsletters or addressing the programme at the start of the day [42].

#### The implementation programme

The IMPROVE programme’s size (large, with many [tailored] implementation activities) and setting (multisite and multiprofessional) appeared to be an important challenge. The IMPROVE study was an ambitious project that aimed to support the implementation of the perioperative safety guidelines in a number of Dutch hospitals. Literature emphasises the importance of keeping a project small, both in terms of the goal(s) and the participants and departments to be included [42]. The perioperative guidelines cover recommendations for the entire perioperative process, from the preoperative screening to the discharge, and relate to continuously changing diverse multiprofessional teams with different powers and cultures and responsible for parts of the perioperative chain.

Especially the hospitals that started later in the stepped-wedge design had to wait long while the feeling of urgency was fed to implement the perioperative guidelines as soon as possible. Hospitals may lose interest or start to implement similar kinds of interventions by themselves [53, 54]. To keep them motivated, it was actually very good that they executed local interventions in the meantime, but this disrupted our (stepped-wedge) design. Besides, this sometimes overloaded the staff participating in project after project. Too many improvement interventions in a short period of time made it difficult to continue a successful programme implementation.

#### Competitive events

Fundamental institutional changes such as mergers and location changes occurred in three hospitals during the study. As a result, priorities shifted in these hospitals. Due to the stepped-wedge design, we were not able to handle this in a flexible manner. More than half of the hospitals were not able to initiate the intervention as scheduled due to factors in the external environment that we were not able to control (such as a formal pause of activities, changes in hospital registration systems that needed a lot of attention and participation in a new accreditation trajectory [55]).

Another competitive event included hospital visits of the Inspectorate (IGJ). The IGJ did not take our study into account. Therefore, hospitals could be visited any time and they put in a lot of efforts to achieve a positive judgement. However, after a positive judgement, the urgency to implement the perioperative guidelines was reduced, resulting in doubts about the added value of the IMPROVE programme.

In conclusion, contextual factors, i.e. anything external to the intervention that may act as a barrier or facilitator to its implementation, or its effects, such as competitive events and co-interventions, need to be taken more systematically and broadly into account when designing an implementation programme. If possible, the intervention should be adapted to the competitive events and it should be accepted that execution of the programme may be delayed [42].

### Process analysis approach

A process evaluation is an essential part of designing and testing complex implementation programmes. Measuring the relationship between the degree to which strategies are implemented as intended and effect can help to distinguish between strategies that are inherently faulty and those that are badly or not at all implemented (implementation failure) [56]. Therefore, we evaluated the degree to which the IMPROVE programme was implemented as intended.

As direct observations cause less socially and personally biased results, we developed and used a new measure to map the involvement of the participating hospitals. The standard process evaluation survey that we developed yielded too little information because of a low response. Therefore, we used our field notes to compare executed and planned activities (i.e. what hospitals actually did based on the field notes compared with what hospitals should have done based on the IMPROVE manual). The structured field notes helped to gain insight in the complex, dynamic and continuously changing real world in which implementation activities must be carried out and to explain the involvement rating measure. The resulting involvement rating measure turned out to be a valuable and robust alternative to the usual PAA, which has been described by Hulscher et al. [22] and the Medical Research Council guidance [57]. By using questionnaires in the target population, “the actual exposure to” and “the experience with” the implementation programme is evaluated [22]. Our field notes yielded more information regarding the content (meetings, presentations; agenda and what has been discussed) and adjustments along the way than the framework of Hulscher et al. [22] could have done. Furthermore, the field notes can be used more objectively (when drafted by an independent researcher instead of by a possibly biased local person) in cases of very low compliance. To our regret, we missed the experience part. This information is important for adaptation or improvement of the programme/intervention.

The involvement rating measure was partially validated by its predictive value, as shown by the correlation with process indicators (Emond et al.: Increased adherence to the national perioperative safety guidelines associated with improved patient safety outcomes. Results of the IMPROVE implementation study, a stepped-wedge, cluster-randomized multicentre trial, submitted). This means that the STOP bundle as well as three (out of six) separate stop moments of the STOP bundle (the time-out, discharge from the recovery ward, and discharge from the hospital) were more successfully implemented in hospitals with a higher involvement rating measure than in the other hospitals. However, we must make a comment on this. For all PSIs we were able to point out best-practice hospitals. But, it seemed difficult for hospitals to achieve high compliance rates on all PSIs; high compliance rates for (a) specific PSI(s) mostly implied average or low compliance rates for (an)other PSI(s).

### Strengths and limitations

A strength of this process evaluation was the development of a new PAA that measures implementation involvement by an involvement rating measures. Based on the field notes, we were able to extract four major implementation challenges and to provide recommendations for future improvement initiatives. To our knowledge, no other PAA has addressed such a wide-range of implementation challenges, offering a very complete and inclusive overview of the challenges that need to be addressed for successful change. The implementation programme was offered in a range of hospitals across the Netherlands representing diverse sizes and teaching statuses, increasing the generalizability of the challenges and subchallenges found.

The framework of van Sluisveld et al. [24] was difficult to apply. The four “main” implementation challenges all fitted in just two categories (i.e. implementation characteristics and institutional characteristics), while some subchallenges fell into a different category than the main challenge concerned. For example, competitive events are challenges related to institutional characteristics, but co-interventions by the Inspectorate are a challenge related to the societal context. The framework turned out to be too unrefined and not suitable to help structure our implementation challenges. It is a framework for barriers on the level of guideline and innovation implementation for target users, but it appeared less usable to specify and differentiate challenges in the execution of an implementation programme to implement guidelines or other innovations.

Moreover, most subchallenges are interrelated and connected with each other (see Additional file 3). For example, the data-collection problems in the participating hospitals became particularly challenging due to the many times that data had to be collected according to the stepped-wedge structure.

Finally, the perspective of this article can be considered as a possible limitation. We have tried to give the best possible representation of the study reality. The whole study was complex and challenging. This is the reason why this manuscript is built on the perspective of the challenges. Two hospitals were an exception. We have tried to capture e.g. what worked in these hospitals in the solutions to the challenges in Table 4. In this way, valuable lessons can be learned from our implementation efforts.

### Practical implications and suggestions for further research

The implementation of the IMPROVE programme was a complex intervention in a strictly hierarchical context. Implementation challenges were multifactorial and affected by considerable clinical, cultural and organizational complexity. In contrast with an explanatory trial, in which an intervention is tested in ideal conditions, a pragmatic trial tests whether an intervention is effective in real life, which in fact was what we did; thus, afterwards, the design proved to have important practical drawbacks, such as the planned time frames of the stepped-wedge design (difficult to maintain), the many necessary repeated measurements and the long trial duration. In addition, we underestimated the efforts needed in our study: some challenges were not anticipated or underestimated in terms of activities or impact. Moreover, we overestimated the commitment of the hospitals and underestimated the step length (of the stepped-wedge design) needed. More time was needed for real changes in patient safety than was anticipated beforehand. The fixed time frame, the overly complex and changing context, with many issues and interests competing for attention, competed with our implementation programme. Programme implementation was also influenced by the size and complexity of the programme. The multicomponent implementation programme with different implementation activities that could be individually tailored to guideline adherence, local barriers and own initiatives turned out to be very time consuming and made this project highly complex.

For the internal support and implementation of innovations, contact persons or internal change agents play an important role. A more thoughtful selection of contact persons per hospital would probably have contributed to the implementation success. The selection of the contact persons should be a firmer condition for participation of a hospital in a study. There should be better agreement about their role and responsibility, in terms of diffusing information (informing employees and bridging information gaps between the top management and employees) and synthesizing information, mediating between strategies and day-to-day activities and motivating colleagues to fill in questionnaires. We advise an application procedure with job interviews and to create a profile with characteristics that a contact person should meet to apply. Based on the positive experiences in some hospitals and scientific literature [58, 59], important characteristics of a contact person should be ability to motivate, connect and enthuse, collaborative leadership, power, social influence, personal connections, being well-respected, credibility, acceptance by the target group, overview, understanding the viewpoints and roles of all stakeholders, and provision of time. Collaborative leadership gives stakeholders a voice in change; provides a clear understanding of the purpose of the department (the core of any culture change); reenergizes the hospital’s vision and values; and helps stakeholders maintain motivated for achieving long-term goals [51]. Informal connections and influence gives contact persons access to opportunities, information, and support, and thus the ability to organize things and mobilize others [59]. Change agents who are central in the hospital’s informal network have a clear advantage, irrespective of their formal hierarchical position as informal networks have been identified as key sources of influence in hospitals [59, 60]. Change agents rely on these informal contacts to build partnerships, shift attitudes towards new ideas and improvement initiatives and overcome resistance to change [60].

We noticed that involvement in the IMPROVE programme was the highest in a small hospital, with a manageable context, more commitment and personal contacts. This is important to realize in advance: the larger the hospital, the longer, the more distant and the more complex the care processes.

A high quality PAA requires good working relationships with all stakeholders. Without good relationships, close observation of the intervention can be challenging, as we saw in our study.

Implementation programmes can fail in terms of outcomes, but by seeing the implementation process with all challenges as a learning process, the research team and other healthcare professionals can learn for future projects.

Future research should strengthen our suggested solutions by enhancing the evidence for these suggestions. Also, linking specific implementation strategies to the challenges we encountered would be a valuable future step. Finally, we recommend that future studies further validate the involvement rating measure.

## Conclusions

The implementation of the perioperative safety guidelines turned out to be a complex intervention. Our PAA shows varying involvement of the participating hospitals in the IMPROVE-implementation programme. The implementation activities were barely carried out in five hospitals, especially in the last groups in the stepped-wedge design. As a standard PAA yielded insufficient information, we developed an involvement rating measure to assess the hospitals’ involvement in the IMPROVE-implementation programme. In cases of very low compliance, an involvement rating measure turned out to be a valuable alternative to the usual PAA, as described by Hulscher et al. [22] and the Medical Research Council [57]. The structured field notes facilitated the explanation of the involvement rating measure. Several implementation-related characteristics explain the implementation gaps, such as: the study design; the implementation programme; and the selection process of the hospitals, the contact persons, the heterogeneous perioperative team and insufficient senior management leadership and support. Co-interventions, competitive events and conflicting priorities also hampered the involvement in the IMPROVE programme. Data needed to measure effects appeared to be a highly underestimated challenge. Study results were based on analysis of information in medical records and routinely collected hospital information system data. Record review is time consuming and may be distorted by missing information. The availability of data depends on the completeness of data entry into applicable fields. For this reason, we recommend investing resources in accurate data registration. Although a stepped-wedge trial design is a powerful design with many benefits, it did not fit well in the concerning study. Hospitals were not always able to fit their implementation activities into their allocated specific time frame; some hospitals wanted the programme sooner and some wanted it later and this discrepancy appeared to be difficult to control. In complex real-life studies, observational designs such as the time series design should be considered.

## Supplementary Information

**Additional file 1.** Outcome measures of the IMPROVE study.

**Additional file 2.** Description of the content of the implementation activities in the IMPROVE standard and additional packages.

**Additional file 3.** Overview of the implementation challenges.

**Additional file 4.** Case example of parallel registration in which the administration time of antibiotic prophylaxis (AB) is documented in three electronic data systems.

**Additional file 5.** This file contains the SQUIRE checklist.

## Data Availability

All data generated or analysed during this study are included in this published article (and its supplementary information files).

## Abbreviations

IMPROVE: Implementation of Perioperative Safety Guidelines, in Dutch: IMPlementatie Richtlijnen Operatieve VEiligheid
IT: Information technologies
PSI: Patient Safety Indicator
PAA: Process analysis approach
IGJ: Dutch Health Care Inspectorate
AB: Antibiotic prophylaxis
OR: Operation room
ICU: Intensive care unit
POWI: Post-operative wound infection
