# Supplementary material for: Complexity and involvement as implementation challenges: results from a process analysis
Source: BMC Health Serv Res. 2021 Oct 23;21:1149. doi: 10.1186/s12913-021-07090-z (PMC8542304; doi:10.1186/s12913-021-07090-z)
Supplement: Supplementary file 1 — Additional file 1. Outcome measures of the IMPROVE study. [file 12913_2021_7090_MOESM1_ESM.docx]

Additional file 1: Outcome measures of the IMPROVE study

| **Primary outcome** (guideline adherence measured by a bundle of nine PSIs on the process and structure of perioperative care**)** |
| --- |
| *Completion of the total STOP bundle (composite of six separate stop moments* in the perioperative care process, i.e. stopping rules that determine whether a patient can go to the next phase in process), % patients*  I. Preoperative risk management – screening the patient  III. Check of the current situation – checking whether all conditions for safe surgery are met  IV. Time-out before the operation  V. Sign-out after the operation  VI. Discharge from the recovery  VII. Discharge from the hospital |
| *Timely administration of AP, % patients* |
| *Availability of a protocol on prophylactic antibiotic use* |
| *Availability of a protocol on anticoagulant use* |
| *Availability of a protocol on responsibilities regarding maintenance of medical equipment* |
| *Availability of a protocol on (performing) prospective risk analysis of medical equipment* |
| *Availability of OR regulations* |
| *Presence of a surveillance system for postoperative wound infections* |
| *Presence of a morbidity and mortality registration* |
| **Secondary outcomes** (patient safety outcomes) |
| *Postoperative wound infections (in-hospital), % patients*  *Postoperative mortality (in-hospital), % patients* The death of a patient during the hospitalisation as the result of any perioperative complication.  *Complications (in-hospital), % patients*  *Length of hospital stay, number of days*  *Unscheduled transfer to the ICU (within 30 days after the initial surgery), % patients* An admission to the ICU department that has not been ordered preoperatively.  *Unscheduled re-operation (within 30 days after the initial surgery), % patients*  *Non-elective hospital-re-admission (within 30 days after the initial surgery), % patients* A re-admission includes at least one overnight stay. Emergency room visits not resulting in hospital admission were not included. |

* Stop II. Surgery planning (date) was excluded because it does not concern direct patient care.
