# Supplementary material for: Complexity and involvement as implementation challenges: results from a process analysis
Source: BMC Health Serv Res. 2021 Oct 23;21:1149. doi: 10.1186/s12913-021-07090-z (PMC8542304; doi:10.1186/s12913-021-07090-z)
Supplement: Supplementary file 2 — Additional file 2. Description of the content of the implementation activities in the IMPROVE standard and additional packages. [file 12913_2021_7090_MOESM2_ESM.docx]

Additional file 2: Description of the content of the implementation activities in the IMPROVE standard

and additional packages

| **Standard package** |
| --- |
| *Small-scale educational meetings*  Workshop or skills training for perioperative key disciplines including assignments, role playing, own presentations, patient stories or discussion and problem solving of hypothetical patient situations/case studies. Provided by an opinion leader within the field of patient safety (*e.g.* one of the medical specialist within the research team) or by a highly respected colleague. Based on active participation in small groups: multi- or mono-disciplinary groups (*i.e.* per discipline, *e.g.* surgeons, recovery nurses, etc. separately). The content is based on the key constraints and most important obstacles in applying the guidelines for a hospital (based on the results of the audit) and a brainstorming session during the training or pre-handed topics that participants find important to discuss. |
| *Audit and feedback*  Feedback is based on the indicator measurement(s), structural observation, barrier analysis and the Team Climate Inventory and Hospital Survey on Patient Safety questionnaires. The feedback consists of a local paper report with the hospital’s own results, benchmarked and presented in relation to all nine participating hospitals. The hospitals in the intervention phase receive this report shortly after a measurement period. Subsequently, the feedback report is presented by the research team and discussed with the key professionals during a meeting. |
| *Structural observation*  Observation by a trained (external) expert of the pre-, per- and postoperative trajectory of one surgical patient (on the ward, operation room and recovery ward) based on a structured observation list. Feedback is based on the completed observation list. The hospitals receive the feedback immediately afterwards. |
| *Local embedding of the guidelines*  Concrete and visible integration into and/or completion of a local protocol and/or checklist. For example, the adaptation of the guidelines in a local protocol, conducting audits (indicator measurements), structural observations and visitation to monitor the implementation of the guidelines, the use of reminder systems (completing existing checklists based on the guidelines; if possible, a new digital checklist may be installed in electronic patient records), decision support and feedback on the implementation of the protocol (using ICT), incorporation of the guidelines in the clinical pathway, *e.g.* resignation letter to the general practitioner. |
| *Patient safety cards*  - Two patient safety cards (with cartoons and explanations) based on the perioperative guidelines, entitled: “Help us with your safe surgery” and “Discharge from the hospital”, are offered to the patients in order to explicitly invite patients to ask questions. |
| **Additional package** |
| *Personal information letter in mailbox*  Personal information letter to all key disciplines about the (use of the) guidelines. |
| *Exchange platform*  A digital platform for the hospitals to exchange best practices, ideas and experiences. |
| *Scan of the total perioperative process*  A practice scan consisting of five parts:  - Hospital staff complete an online survey about the perioperative process  - Interviews with hospital staff (more background information regarding remarkable survey answers)  - Structured observation on site - Paper report by post (contains findings, a top-5 of strengths and weaknesses and recommendations)  - Feedback meeting to discuss the report. |
| *Posters*  Visual representation of, for example, the perioperative trajectory of the patient with the stop moments, shown as a subway line.  *Electronic reminder message*  Catchy quotes on behalf of an opinion leader within the perioperative process of a hospital. The content is based on detected bottlenecks for the hospital. |
| *Multiprofessional team training*  The IMPROVE team facilitates contacts between the participating hospitals and organizations that provide training programmes aimed at improving team culture, such as crew resource management. |
