# Supplementary figures and images for: Complexity and involvement as implementation challenges: results from a process analysis
Source: BMC Health Serv Res. 2021 Oct 23;21:1149. doi: 10.1186/s12913-021-07090-z (PMC8542304; doi:10.1186/s12913-021-07090-z)

Additional file 3: Overview of the implementation challenges


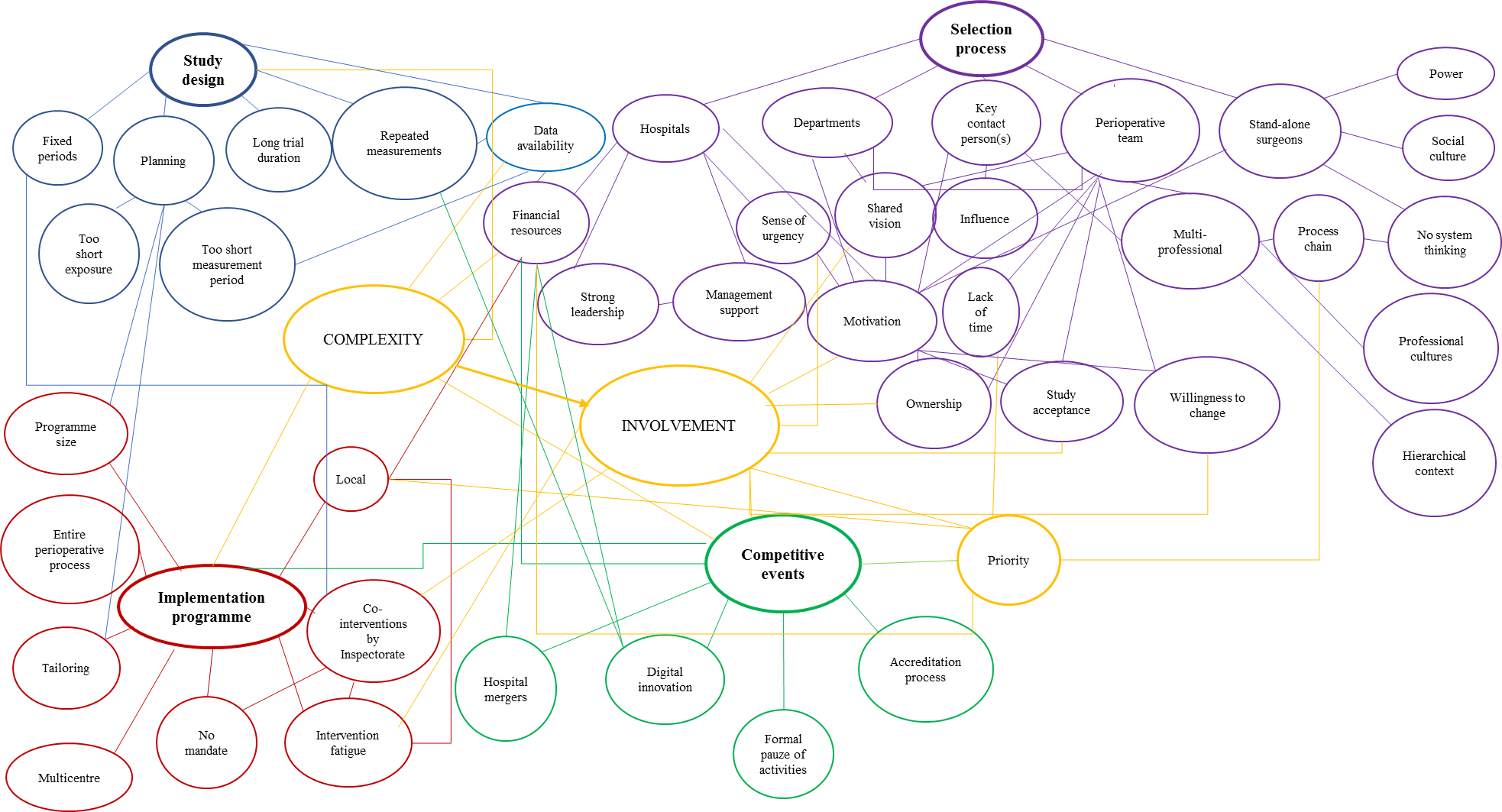

Supplement: Supplementary file 3 — Additional file 3. Overview of the implementation challenges. [file 12913_2021_7090_MOESM3_ESM.docx]
