# Supplementary material for: Complexity and involvement as implementation challenges: results from a process analysis
Source: BMC Health Serv Res. 2021 Oct 23;21:1149. doi: 10.1186/s12913-021-07090-z (PMC8542304; doi:10.1186/s12913-021-07090-z)
Supplement: Supplementary file 4 — Additional file 4. Case example of parallel registration in which the administration time of antibiotic prophylaxis (AB) is documented in three electronic data systems. [file 12913_2021_7090_MOESM4_ESM.docx]

Additional file 4: Case example of parallel registration in which the administration time of antibiotic prophylaxis (AB) is documented in three electronic data systems
